# Application of *Lactobacillus plantarum* and *Pediococcus lactis* on Lipid Metabolism, Anti-Inflammatory, and Fecal Microbiota in Cats

**DOI:** 10.3390/microorganisms12122446

**Published:** 2024-11-28

**Authors:** Shukun Liang, Xinshu Gu, Jintao Sun, Xiumin Wang, Hui Tao, Zhenlong Wang, Yougang Zhong, Jinquan Wang, Bing Han

**Affiliations:** 1 Key Laboratory of Feed Biotechnology of Ministry of Agriculture and Rural Affairs, Institute of Feed Research, Chinese Academy of Agricultural Sciences, No. 12 Zhong Guan Cun South Street, Haidian District, Beijing 100081, China; 18811536925@163.com (S.L.); 15957544121@163.com (X.G.); 82101235453@caas.cn (J.S.); wangxiumin@caas.cn (X.W.); taohui@caas.cn (H.T.); wangzhenlong01@caas.cn (Z.W.); 2 School of Veterinary Medicine, China Agricultural University, Beijing 100193, China; zhongyougang@126.com; 3 College of Animal Science and Technology, Jiangxi Agricultural University, No. 1225, Zhimin Avenue, Xinjian District, Nanchang 330045, China

**Keywords:** lactic acid bacteria, cats, lipid metabolism, fecal microbiota

## Abstract

Probiotics have been used in functional foods and dietary supplements, and in recent years, they have become more widely used in pets. In our previous experiment, *Lactobacillus plantarum* L-27-2 and *Pediococcus lactis* L-14-1 were isolated from cat feces and proved to have positive effects on lipid metabolism in mice. To further discuss their possible effects in cats, a total of 12 healthy cats (British Shorthair) were randomly divided into two groups. One group was fed *Pediococcus lactis* L-14-1 (1 × 10^9^ CFU/kg/d, n = 6), and the other group was fed *Lactobacillus plantarum* L-27-2 (1 × 10^9^ CFU/kg/d, n = 6), and the experiment was conducted for 28 days. Blood and feces were collected on days 0 and 28 separately. ELISA was used to detect blood biochemical indexes in cats. The results showed that L-27-2 and L-14-1 could reduce the content of TG (triglyceride, *p* < 0.05) and LDL-C (low-density lipoprotein cholesterol, *p* < 0.01) in the blood, increase the content of HDL-C (high-density lipoprotein, *p* < 0.01), and L-27-2 could significantly reduce the content of IL-6 (*p* < 0.01). The diversity of feces microbiota was also tested. On the phylum level, there was no significance in the phylum level of Firmicutes and Bacteroidetes (*p* > 0.05), but on the genus level, in the L-14-1 group, the abundance of *Lantiplantibacillus* and *Cetobacterium* was increased (*p* < 0.05), and the abundance of *Ruminococcus*, *Olsenella*, and *Labanicoccus* was decreased (*p* < 0.05), while in the L-27-2 group, the abundance of *Libanicoccus* was also decreased in L-14-1 (*p* < 0.05). Above all, L-27-2 and L-14-1 can be considered potential probiotics to improve cat gut health and lipid metabolism.

## 1. Introduction

In recent years, gut health has become more important for pets. Recent efforts have focused on certain functional food probiotics, which are live microorganisms, showing beneficial effects on the gut health of the host [1]. Lactic acid bacteria are widely used on humans and livestock, many species of which could improve nutrient absorption and gut function, modulate lipid metabolism and obesity, inhibit the growth of pathogens, and even improve immune function [2,3,4,5].

Obesity, causing many diseases in pets, is a serious problem, and it is increasing [6], especially in the elderly. The obesity problem was closely related to the lipid metabolism of pets. Adipose tissue, recognized as an endocrine organ, actively participates in carbohydrate and lipid metabolism, energy regulation, and the inflammatory and coagulation cascades [7]. Some studies have shown that probiotics regulate the mechanism of lipid metabolism [8], reduce animal weight and liver health, improve blood glucose levels, and reduce adipose tissue weight [9,10]. With the improvement of the living levels of humans and pets, obesity poses an increasing risk to the health of cats; the studies of obesity have expanded from humans to cats [11,12,13].

An imbalance in the gut microbiota is associated with obesity, with one study demonstrating that the gut microbiota of overweight and obese cats is significantly different compared to lean cats [14]. The addition of *Lactobacillus acidophilus* D2/CSL (CECT 4529) could also improve fecal quality in healthy cats, increase the number of *Lactobacillus*, and reduce the number of *Escherichia coli*, thereby enhancing gut health [15]. However, it is worth noting that the research on the effects of probiotics on overweight and obese cats is not adequate. In a recent study, probiotics had no significant effect on food intake, body weight, and metabolic parameters in overweight and obese cats [16]. Therefore, further studies are needed to evaluate different types and strains of probiotics and verify the effects of probiotics on lipid metabolism and gut microbiota in cats.

Probiotics began to be applied in cats in recent years. The previous studies showed that after feeding constipated cats with the multi-strain probiotic SLAB51^TM^, containing *Lactobacilli*, *Bifidobacteria*, and *Streptococcus* species, the abundance of *Lactobacillus* spp. (*p* = 0.03) and *Bacteroidetes* (*p* < 0.05) was increased, and the symptoms of constipation and potential anti-inflammatory effects were markedly improved [17]. Also, the use of multi-strain probiotics could promote gut health by modulating gut microbes, improving microbiota-derived short-chain fatty acid production, reducing inflammation, and improving antioxidant status in healthy, short-haired domestic cats [18], which showed that probiotics could probably be beneficial for cats. In previous studies, microbiota were focused on, and lipid metabolism was less studied at the same time. In our previous research, it has been proved that *Lactobacillus plantarum* L-27-2 and *Pediococcus lactis* L-14-1 isolated from cats’ feces have positive effects on decreasing cholesterol and inhibiting pathogenic bacteria in mice [19].

In this study, in order to observe the effect of the two strains on healthy cats, we first fed the two strains to healthy cats, aiming to evaluate the effects of the two isolates on the blood biochemistry and fecal microbiota of adult cats. Also, provide more evidence for the safer use of these strains in cat clinics.

## 2. Materials and Methods

### 2.1. Preparation of Lyophilized Lactic Acid Bacteria

In our previous research, two newly isolated strains, *Lactobacillus plantarum* L-27-2 (hereinafter referred to as L-27-2) and *Pediococcus lactis* L-14-1 (hereinafter referred to as L-14-1), were isolated from healthy cat feces [19], which were deposited in the General Microbiology Center of the China Microbial Culture Collection Administration Committee with the deposit numbers CGMCC No. 27193 and CGMCC No. 27676, respectively.

For functional foods containing probiotics to be eligible to make health claims, microorganisms must be able to resist the processing operations, handling, storage, and finally, the passage through the gastrointestinal tract [20]. In order to protect probiotics, a series of processes such as processing, storage, and digestion will cause probiotics to lose their vitality, among which freeze-drying is an indispensable and valuable preservation method to ensure the long-term stability of bioactive products and has become a reference process for the preservation of lactic acid bacteria [21]. So, in this experiment, L-27-2 and L-14-1 were freeze-dried. L-27-2 and L-14-1 were inoculated at 1% inoculum in MRS (Solarbio, Beijing, China) and cultured at 37 °C for 24 h. L-27-2 and L-14-1 were fermented (MRS liquid medium, 37 °C, 48 h) and concentrated (6000× *g*/min, 10 min), and then lyophilized adult powder (10^12^ CFU/g) with a lyophilizer (Sihuan Bioengineering Co., Ltd., Beijing, China).

### 2.2. Animals and Experimental Design

The animal test was implemented according to the Animal Care and Use Committee of the Institute of Feed Research of the Chinese Academy of Agricultural Sciences (CAAS) and was approved by the Laboratory Animal Ethical Committee and its inspection of the Institute of Feed Research of CAAS (IFR-CAAS-20231027).

In this study, 12 healthy adult cats (British shorthair, aged 2–5 years) with normal weight (2–4 kg) were selected and obtained from Tianjin Cat Experimental Base. All cats were not treated with antibiotics prior to the trial. During the experiment, they were kept separately in cages (length: 162 cm; width: 62 cm; and height: 62 cm); only staple food and lactic acid bacteria were fed, without any other food, and all cats were fed by the ad libitum feeding method, with no restriction on drinking water, and no antibiotics were used during the test.

Twelve cats were randomly divided into two groups, with six cats in each group (male/female = 2:4). A within-subject study design was used to remove an important source of between-subject variation, with each cat representing its own control [22], and L-27-2 and L-14-1 lyophilized bacterial powders were administered to the L-27-2 group and L-14-1 group for 28 days continuously, and the dose was 1 × 10^9^ CFU/kg/d, and the lyophilized bacterial powders were thoroughly stirred and mixed with the cat’s basal diet (100 g) every day and then fed. All cats were free to eat and drink. The fecal and blood samples from the cat were collected.

### 2.3. Detection of Blood Lipid-Related Indexes and Inflammation-Related Indicators

Blood serum was taken for analysis on days 0 and 28. Approximately 1–1.5 mL of blood is collected from the cat’s saphenous vein by venipuncture. After the whole blood was naturally coagulated and precipitated into a light yellow and bright liquid, the serum was prepared by centrifugation at about 2000× *g* for 10 min at 4 °C, and ELISA (Jiangsu Meimian Industrial Co., Ltd., Yancheng, China) was used to detect inflammatory cytokines, including IL-6 (interleukin-6), TNF-α (tumor necrosis factor-alpha), and lipid metabolism-related indicators, including TC (total cholesterol), TG (triglyceride), HDL-C (high-density lipoprotein cholesterol), and LDL-C (low-density lipoprotein cholesterol).

### 2.4. Extraction of Fecal DNA

Feces collection is performed on days 0 and 28. All feces were collected immediately after defecation using clean, sterile 50 mL centrifuge tubes. Part of the feces was divided into sterile and clean 2 mL centrifuge tubes and stored in a −80 °C refrigerator for DNA extraction. Microbial genomic DNA was extracted from cats’ fecal samples using the E.Z.N.A. Mag-Bind Soil DNA Kit (Omega, M5635-02, San Antonio, TX, USA), and the concentration of the DNA samples was detected with the Quibit dsDNA HS kit (Thermo, Waltham, MA, USA).

### 2.5. PCR Amplification

PCR products were detected by electrophoresis. The V3-V4 region of the 16S rDNA was amplified and sequenced by second-generation sequencing technology at Sangon Biotech Co., Ltd. (Shanghai, China).

The sequence of the forward primer was CCTACGGGNGGCWGCAG, while the reverse primer was GACTACHVGGGTATCTAATCC. PCR testing was amplified twice. The first PCR reaction conditions referred to the method [23]. The amplification procedure was as follows: pre-denaturation at 95 °C for 3 min, 27 cycles (denaturation at 95 °C for 30 s, annealing at 55 °C for 30 s, extension at 72 °C for 30 s), followed by stable extension at 72 °C for 10 min, and finally storage at 4 °C.

### 2.6. Library Construction and Hands-On Sequencing

A total of 1 μg of genomic DNA was taken, and the fragments with a length of about 350 bp were randomly interrupted by the Covaris ultrasonic breaker, and the library was constructed, and the whole library was prepared by end repair, A-tailing, sequencing adapter, purification, PCR amplification, etc. Once the library was constructed, it was initially quantified using Qubit 2.0, diluted to 2 ng/μL, and then the insert size of the library was detected using the Agilent 2100, and the effective concentration of the library was accurately quantified using Q-PCR (>3 nM) to ensure library quality. After passing the library inspection, Illumina PE150 sequencing was performed after pooling different libraries according to the requirements of effective concentration and target data volume.

### 2.7. Data Analysis

Principal coordinate analysis (PCoA) was used to determine beta diversity, which adopted unweighted UniFrac. The alpha diversity index was tested by T-test and Wilcox rank sum test to determine whether there were significant differences in species diversity between groups. Differences were compared using STAMP version 2.1.3 and LefSe (version 1.1.0) software to identify features with significantly different abundances between groups. In addition to the 16S rRNA data described, additional data obtained in this study were performed using IBM SPSS statistical software (version 19.0, IBM, Armonk, NY, USA), and a one-way analysis of variance (ANOVA) was used for differences between more than two groups, followed by a Newman–Keuls post hoc test.

It should be noted that *p* < 0.05 is considered statistically significant. Use the following *p*-values: * *p* < 0.05, ** *p* < 0.01.

## 3. Results

### 3.1. Blood Biochemistry

**Figure 1:**
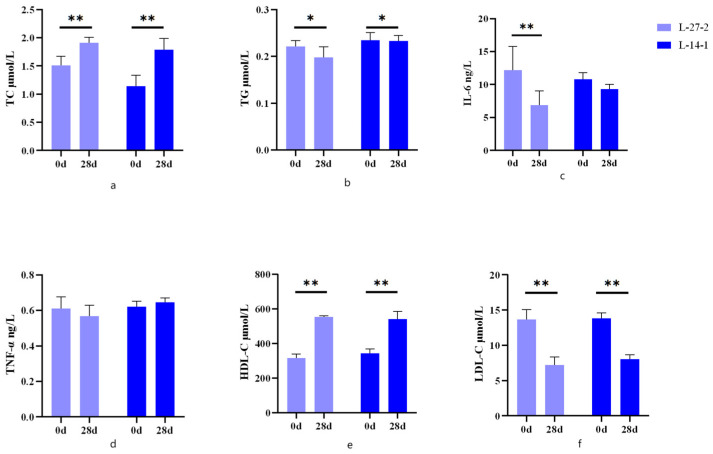
Changes in blood chemistry on days 0 and 28 in cats. (**a**) serum TC content; (**b**) serum TG content; (**c**) serum IL-6 content; (**d**) serum TNF-α content; (**e**) serum HDL-C content; and (**f**) serum LDL-C content. * *p* < 0.05. ** *p* < 0.01.

Both L-14-1 and L-27-2 could significantly increase the contents of TC and HDL-C (*p* < 0.01) (Figure 1a,e), decrease the content of LDL-C (*p* < 0.01) (Figure 1f), and decrease the content of TG (*p* < 0.05) (Figure 1b). Detection of inflammatory cytokines in the blood showed that L-27-2 significantly reduced IL-6 levels (*p* < 0.01) (Figure 1c). There was no significant effect on TNF-α content in either bacteria (*p* > 0.05) (Figure 1d).

These results showed that both strains could cause changes in blood lipids in healthy cats.

### 3.2. Fecal Microbiota

**Figure 2:**
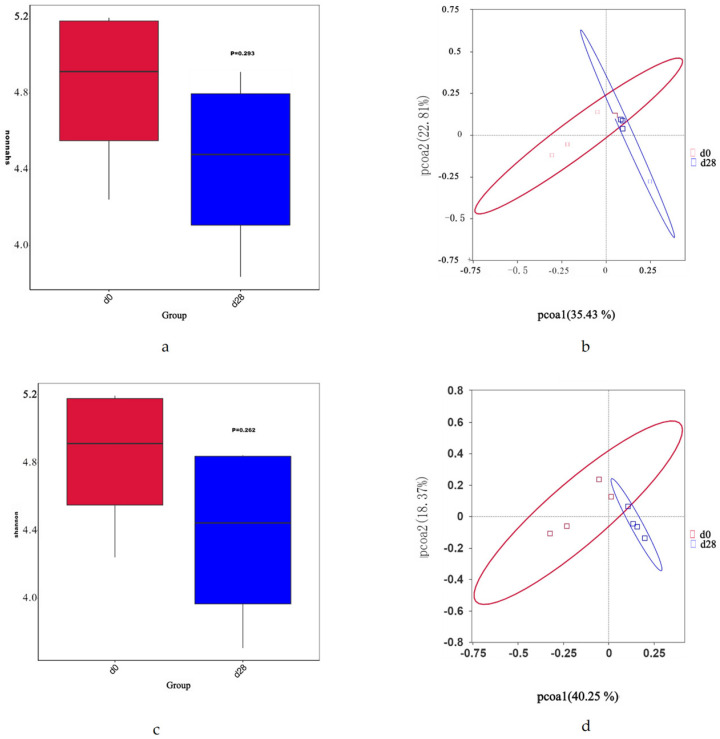
Fecal Microbiota Analysis. (**a**) α-diversity (Shannon index) in the L-14-1 group; (**b**) PCoA analysis of the L-14-1 group; (**c**) α-diversity (Shannon index) in the L-27-2 group; and (**d**) PCoA analysis of the L-27-2 group.

There was no significant difference in the diversity of fecal microbiota on the 28th day compared with day 0 in both groups for both isolates (*p* > 0.05) (Figure 2), but the overall trend program showed a downward trend.

**Figure 3:**
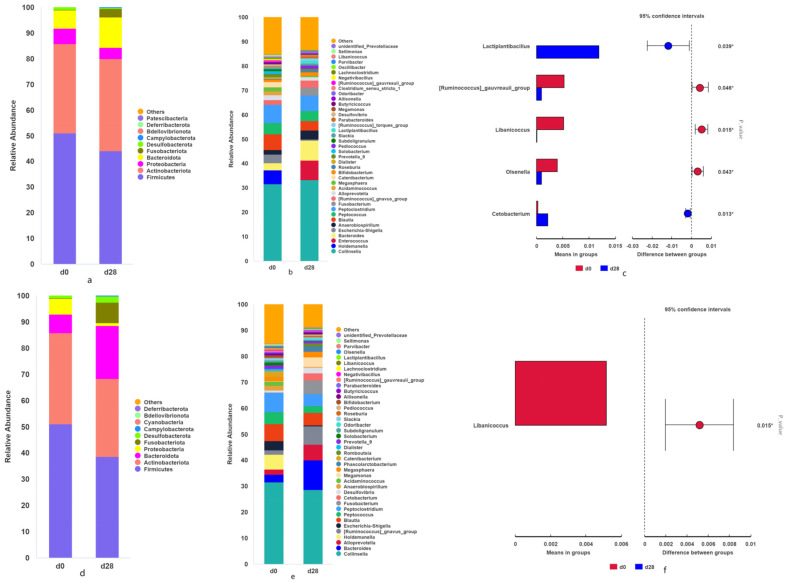
(**a**) Abundance of phylum level of fecal microbiota on day 0 and 28 of the L-14-1 group; (**b**) abundance of genus level of fecal microbiota on day 0 and 28 of the L-14-1 group; (**c**) significance of the genus differences in the L-14-1 group; (**d**) abundance of phylum level of fecal microbiota on day 0 and 28 of the L-27-2 group; (**e**) abundance of genus level of fecal microbiota on day 0 and 28 of the L-27-2 group; and (**f**) significance of the genus differences in the L-27-2 group.* *p* < 0.05.

There were significant differences in five genera on day 28 compared to day 0 after using L-14-1, namely *Lactiplantibacillus* (*p* = 0.039), *Ruminococcus-gauvreauii-group* (*p =* 0.046), *Libanicoccus* (*p* = 0.015), *Olsenella* (*p* = 0.044), *Cetobacterium* (*p* = 0.013), and *Fournierella* (*p* = 0.049) (Figure 3a–c). In the L-27-2 group, the abundance of *Libanicoccus* (*p* = 0.014) in the fecal microbiota of the 28th trolls was significantly reduced compared to day 0 (Figure 3d,e). On the genus level, in both groups, *Libanicoccus* was significantly lower in the intestinal microbiota on the 28th day than on day 0 (Figure 3f).

## 4. Discussion

### 4.1. Lactobacillus plantarum and Pediococcus lactis Reduced Blood Lipid Levels

Probiotics have been increasingly used in companion animals, and current evidence suggests that probiotics have a beneficial role in promoting health and disease prevention in dogs and cats. However, it remains underexplored, particularly with regard to lipid metabolism, when compared to research on humans and livestock [24,25,26]. To date, there have been few studies on the use of probiotics on lipid metabolism in cats, and the efficacy of probiotics in cats cannot be inferred from studies in dogs due to differences in host physiology and diet [22]. Therefore, we used the strains screened from cat feces to do the experiment.

In our former studies, it has been proved that dyslipidemia, gut microbiome, and liver metabolism in rats with a high-fat diet (HFD) induced hyperlipidemia can be modulated by feeding *Lactobacillus plantarum* [19]. Increases in TC, TG, and LDL-C were strongly associated with hyperlipidemia, obesity, and other diseases [27]. Obesity is often associated with dyslipidemia, such as elevated TC, TG, and LDL-C levels and decreased HDL-C levels [28]. Therefore, the serum levels of TC, TG, LDL-C, and HDL-C in cats were measured after 28 days of feeding to assess their lipid metabolism. In this study, two probiotics, *Pediococcus lactis* L-14-1 and *Lactobacillus plantarum* L-27-2, had significant effects on TG, HDL-C, and LDL-C of cats, and the mechanisms of these two probiotics on lipid modulation need further validation.

### 4.2. Lactobacillus plantarum and Pediococcus lactis Reduced the Levels of Blood Inflammatory Factors

Previous studies showed that *Lactobacillus plantarum* and *Pediococcus lactis* had a certain beneficial effect in cats, which could improve the intestinal health and immune function of cats to a certain extent [19,27,29,30]. Studies have shown that *Lactococcus* can promote the development of the immune system, enhance antioxidant capacity, increase the diversity of intestinal microorganisms, and increase the abundance of lactic acid bacteria [31,32]. Compared with day 0, the pro-inflammatory factor (IL-6) of *Lactobacillus plantarum* L-27-2 was significantly reduced after 28 days, and its effect on the treatment of inflammation had a certain effect, but the specific treatment effect still needs to be further studied.

### 4.3. Lactobacillus plantarum and Pediococcus lactis Altered the Microbial Composition of Cat Feces

In this study, the microbiota in the feces of cats were mainly Actinomycetes and Firmicutes at the phylum level. The fecal microbiota of healthy cats is mainly Firmicutes, Proteobacteria, Bacteroidetes, Fusobacteria, and Actinobacteria [26,33]. In the L-14-1 group, the abundance of *Ruminococcus-gauvreauii*-group, *Libanicoccus*, and *Fournierella* was significantly decreased on day 28 compared to day 0, and the abundance of *Libanicoccus* on day 28 was significantly decreased in the two groups on day 28. Studies have found that the *Ruminococcus_gauvreauii*_group and *Fournierella* are associated with metabolic disorders and obesity [34,35,36]. *Cetobacterium* has been studied in the gut microbiota of fish and is generally used to improve glucose homeostasis and increase insulin expression [37]. As an anaerobic bacterium, *Cetobacterium* can participate in various metabolic activities, synthesize vitamins [38], reduce intestinal inflammation, and increase the body’s antiviral ability [39]. This experiment proved that *Lactobacillus plantarum* L-14-1 had a better colonization effect in cats, and at the same time, it increased the abundance of *Cetobacterium* and *Lactiplantibacillus*, the core beneficial bacteria in the gut, and exerted more probiotic effects.

According to the results of intestinal microbiota analysis, the changes in fecal microbiota on day 0 and day 28 of the two groups had a certain correlation with the blood biochemical results, and the probiotic effect of *Pediococcus lactis* L-14-1 on the fecal microbiota and lipid metabolism of cats was more obvious.

In summary, we found that both L-27-2 and L-14-1 can influence blood lipid levels, inflammatory markers, and the microbial composition of cat feces. In this experiment, 12 cats were used with a pre-and post-test design, which helped reduce the impact of individual variability and improved the reliability of the results. However, with only 6 samples per group, the limited sample size restricted our ability to conduct more in-depth statistical analysis and further exploration of the data. Future studies will aim to increase the sample size to enable more comprehensive analyses, such as metabolomics, to better understand the underlying mechanisms.

## 5. Conclusions

This experiment demonstrated that L-27-2 and L-14-1 have potential probiotic effects on inflammation, lipid metabolism, and fecal microbiota in cats. These findings suggest that these strains may offer a promising approach to improving cat health, particularly in terms of gut health and obesity management. Future research should focus on exploring the underlying mechanisms through techniques such as metabolomics and genomic analysis, as well as evaluating the long-term effects of these probiotics.

## Data Availability

The data presented in this study are available upon request from the corresponding author.
